# Mobile Health to Support Community-Integration of Individuals With Disabilities Using iMHere 2.0: Focus Group Study

**DOI:** 10.2196/31376

**Published:** 2022-03-04

**Authors:** Rebecca E Ward, I Made Agus Setiawan, Eleanor Quinby, Melva Fair, Zara Ambadar, Bambang Parmanto, Brad E Dicianno

**Affiliations:** 1 Department of Physical Medicine and Rehabilitation School of Medicine University of Pittsburgh Pittsburgh, PA United States; 2 Department of Health Information Management School of Health and Rehabilitation Sciences University of Pittsburgh Pittsburgh, PA United States; 3 Department of Computer Science Udayana University Badung, Bali Indonesia; 4 Human Engineering Research Laboratories Veterans Affairs Pittsburgh Healthcare System Pittsburgh, PA United States; 5 Community Living and Support Services Pittsburgh, PA United States

**Keywords:** community integration, self-care, mobile health, smartphone, rehabilitation, disability, mobile phone

## Abstract

**Background:**

Mobile health (mHealth) systems that support self-management can improve medical, functional, and psychosocial outcomes for individuals with disabilities and chronic conditions. The mHealth systems can potentially be expanded to support community integration.

**Objective:**

The purposes of this study were to (1) partner with a community-based organization that supports community integration of individuals with disabilities; (2) identify software requirements needed to support community participation; and (3) iteratively refine an existing mHealth application to include new requirements.

**Methods:**

Community Living and Support Services (CLASS), a nonprofit organization that serves individuals with disabilities in Pittsburgh, Pennsylvania, was identified as the focus group for this study. Key stakeholders within the Community Partners Program at CLASS proposed design requirements for an existing mHealth application, Interactive Mobile Health and Rehabilitation (iMHere) 2.0, that has been used to support self-management.

**Results:**

We gathered qualitative data from a focus group composed of CLASS members to develop and iteratively revise iMHere 2.0 to include new modules and features to support community integration. A caregiver app was also developed. The new system contains features to support finance, transportation, client and caregiver communication, calendar and checklist management, upcoming medical and nonmedical appointments, social engagement, pain management, and access to a personal profile. Modifications were made to the following existing modules: education, mood, personal health record, goals, medications, and nutrition.

**Conclusions:**

A successful partnership with a community-based organization that supports individuals with disabilities resulted in a newly designed mHealth system with features to support community integration.

## Introduction

Living in the community affords many benefits for individuals with disabilities and chronic conditions. Studies have shown that higher integration into the community is associated with better health outcomes, longevity, higher quality of life, and lower cost of care [1-3]. However, many barriers to community living exist, many of which are due to fragmented outpatient, inpatient, long-term, and community-based services operating under different funding streams and regulations. This system isolates individuals, leaves many medical and social needs unmet, and ultimately drives costs to enormous and unsustainable levels [4]. It has been proposed that software-based tools—specifically mobile health (mHealth) systems—may be able to improve the process [5].

### Background

Interactive Mobile Health and Rehabilitation (iMHere) is an mHealth system designed to help individuals, including those with disabilities, manage chronic and complex medical conditions so that they can live independently and integrate more fully into the community. The original version of iMHere (iMHere 1.0) focused primarily on self-management tasks aimed to prevent medical complications and promote health. Five modules were developed to allow the user to manage skin care, medications, bladder self-catheterization, bowel programs, and mental health [6]. Through these modules, users could indicate completion of self-management tasks, send adherence data to a portal monitored by a clinician, and receive personalized regimens from a clinician. By providing a secure connection between a client’s smartphone and a web-based clinician portal, iMHere supported Health Insurance Portability and Accountability Act–compliant messaging between a client and team of clinicians [7]. The portal categorized and organized client responses in a dashboard that triaged and flagged data based on urgency.

### Prior Work

Preliminary research with iMHere 1.0 includes a series of focus groups, usability studies, accessibility studies, and clinical trials. Focus groups and usability studies have driven an iterative, user-centered design process and demonstrated that individuals desire an app that is easy to use and engaging, that can provide educational materials, motivation, and support, and that can be personalized [8-13]. Accessibility studies resulted in improvements to accessibility for persons with motor, sensory, and cognitive impairments [14-16]. A clinical trial demonstrated that individuals with spina bifida who were frequent users of iMHere showed positive changes in self-management as well as reduced need for caregiver support [17]. iMHere also demonstrated potential for monitoring and preventing skin wounds in individuals with spina bifida [18]. A recent study found that the iMHere mHealth system aided in the prevention of urinary tract infections and reduction in depression symptoms in individuals with spinal cord injury [19].

Based on this prior research, iMHere 2.0 was developed [11]. The new features include dual iOS and Android platforms, new accessibility and personalization features, a reward center, and 7 additional modules (education, personal health record [PHR], goal setting, wheelchair maintenance, exercise, nutrition, and supplies). Further, a caregiver app allows family members, legal guardians, and attendant care providers to monitor the client’s activity and provide encouragement.

### Study Goal

The primary aim of this project was to expand iMHere 2.0’s functionality to support a community-based organization that assists individuals with disabilities and encourages independent living. To live independently in the community, individuals with disabilities and chronic conditions often need services and supports provided by community-based organizations. We partnered with a community-based organization that fosters community inclusion for individuals with disabilities. Located in the East End of Pittsburgh, Pennsylvania, Community Living and Support Services (CLASS) is a nonprofit organization that offers a variety of individualized services such as community-based case management for social, recreational, and residential supports for individuals with disabilities. This manuscript describes the iterative development process of the features created for iMHere to support services offered by CLASS through the examination of qualitative data obtained from a focus group.

## Methods

The study was approved by the University of Pittsburgh Institutional Review Board (STUDY20020049), and all participants underwent an informed consent process. Key stakeholders who oversee the Community Partners Program (CPP) at CLASS, an initiative that assists individuals with disabilities in both their homes and communities, served as this study’s focus group.

Individuals with disabilities enrolled in the CPP choose specific goals and develop strategies to meet their needs. This program provides one-on-one support. Each client is assigned a case manager who supports short- or long-term goals based on the individual’s defined needs. The CPP emphasizes community integration and strives to reduce the amount of both medical and nonmedical services required by clients. Further, the CPP can partner with individuals to foster connections with community resources, assist with decision-making and problem-solving, impart compensatory strategies, help in the search for employment and volunteer opportunities, and provide life skills training.

With the aims of the CPP in mind, we met with the CPP stakeholders on January 26, 2016, and July 6, 2017, to discuss how iMHere 2.0 could best support a community-based organization and to subsequently develop design criteria. At these initial focus group meetings, stakeholders were given a demonstration of how iMHere 2.0 functions. Stakeholders then presented the documents and forms used by case managers and other employees to manage a client’s case. Information from these documents was organized into themes and used to develop a mock-up for a new design of iMHere. This mock-up was presented to the stakeholders on March 26, 2018. Feedback was gathered and subsequent changes to iMHere 2.0 were made and presented again on April 5, 2019.

## Results

### Overview

A focus group of 15 stakeholders participated in meetings. The demographics of the stakeholders are presented in Table 1 to illustrate the diversity of the participants and the roles that they would play in using the iMHere 2.0 system. The participants represented a variety of roles at CLASS and had different levels of interaction with clients. The focus group was mainly composed of participants who had a professional relationship with the client. Table 2 outlines the demographics of the clients who received services from the focus group participants. Some stakeholders were assigned to more than one client and, therefore, played more than one role.

**Figure 1:**
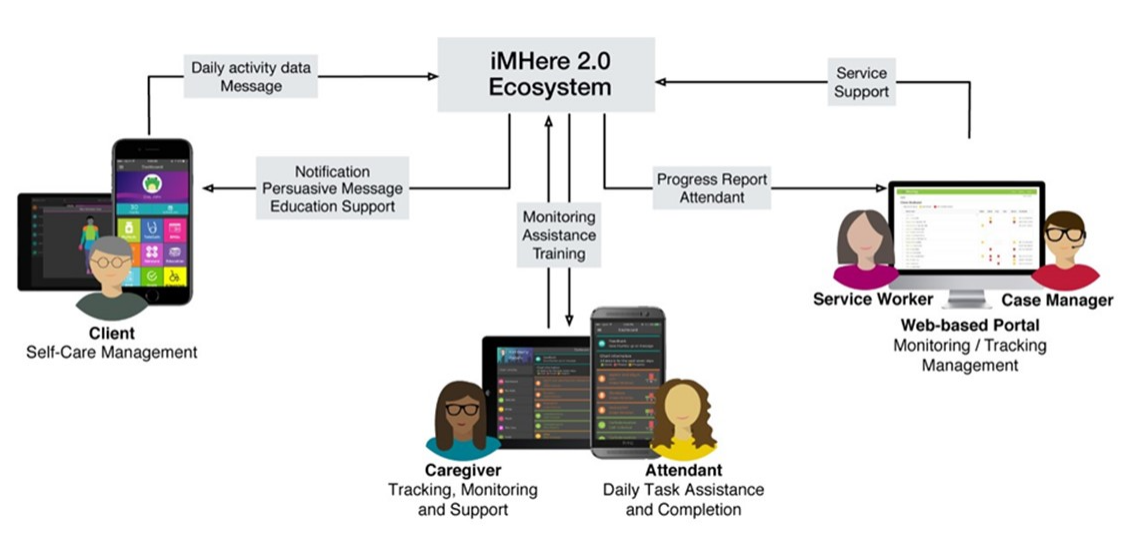
Overview of the iMHere 2.0 system.

Stakeholders identified 5 roles for iMHere 2.0 users based on existing CPP workflow. The “client” is defined as the person receiving services through CLASS. These users would have access to the client app. They can receive services from one or more of the care roles. “Case managers” include program directors and those who oversee the 3 other care roles. Case managers would be web portal users with the highest level of access to the system. “Service workers” include community partners who work with the client to develop and meet goals regarding self-care. Service workers oversee attendants and directly track client activity. These users would have access to both the web portal and the caregiver app. “Caregivers” are those who provide assistance to the client in an unpaid role and would have access to the caregiver app. This category can include family members or friends who provide direct care to clients and need to directly track client activities or ensure that clients complete daily tasks. The “attendant” role is defined as a paid, direct care worker who needs to keep a record of the assigned client’s completed tasks. Attendants would have access only to the caregiver app, with an attendant-specific module. Attendants can serve more than one client and are expected to use the app primarily for recording task completion, which can be approved by the client. Figure 1 shows how users within each role would utilize the 3 frontend components of iMHere 2.0: Web Portal, Client App, and Caregiver App.

Stakeholders requested many new features for iMHere 2.0 (Multimedia Appendix 1). The qualitative data collected from the focus group were bundled into common themes. Features with higher priority (necessary) were developed for the current system, while those with lower priority (desired) were set as future design criteria. Some new features were implemented as additions to existing modules or the portal, while others were built as new modules or portal features.

**Table 1:** Demographics of focus group participants (N=15).

Characteristic			n
**Age (years)**			
	25-34		5
	35-44		1
	45-54		7
	55-64		1
	65-74		1
**Gender**			
	Female		14
	Male		1
**Race**			
	White/Caucasian		14
	Black/African American		1
**Ethnicity**			
	Hispanic/Latinx		13
	Non-Hispanic/not Latinx		2
**Household income**			
	25,000 or less	1	
	26,000-50,000	6	
	51,000-75,000	3	
	76,000-100,000	1	
	101,000-125,000	1	
	More than 125,000	1	
	Declined to answer	2	
**Community of residence**			
	Suburban		13
	Urban		1
	Rural		1
**Education level**			
	High school diploma		2
	Trade school diploma		1
	Associates degree		5
	Bachelor’s degree		6
	Master’s degree		1
**Marital status**			
	Married/long-term relationship		10
**Expected role in the iMHere^a^ system (some participants had more than one role)**			
	Portal user-administrator (technical support or training)		3
	Portal user-service worker		7
	Portal user-speech language pathologist		1
	Portal user-case manager		5
	CG app user-direct care provider/attendant care worker		4

^a^iMHere: Interactive Mobile Health and Rehabilitation.

**Table 2:** Demographics of clients served by stakeholders.

Characteristic		n		
**Clients served**				
	Male clients		6	
	Female clients		6	
	Both male and female clients		3	
**Client age (years)**				
	18-24	2		
	25-34	2		
	35-44	2		
	45-54	4		
	55-64	8		
	65-74	0		
	≥75	1		
**Client diagnosis**				
	Cerebral palsy		8	
	Traumatic brain injury		5	
	Spina bifida		2	
	Spinal cord injury		1	
	Autism spectrum disorder/intellectual disability		1	
	Diabetes and heart disease		1	
**Client secondary diagnosis**				
	Medical	3		
	Mental health	2		
	Learning disability	1		
	Multiple	3		
	None	6		

### Caregiver App

**Figure 2:**
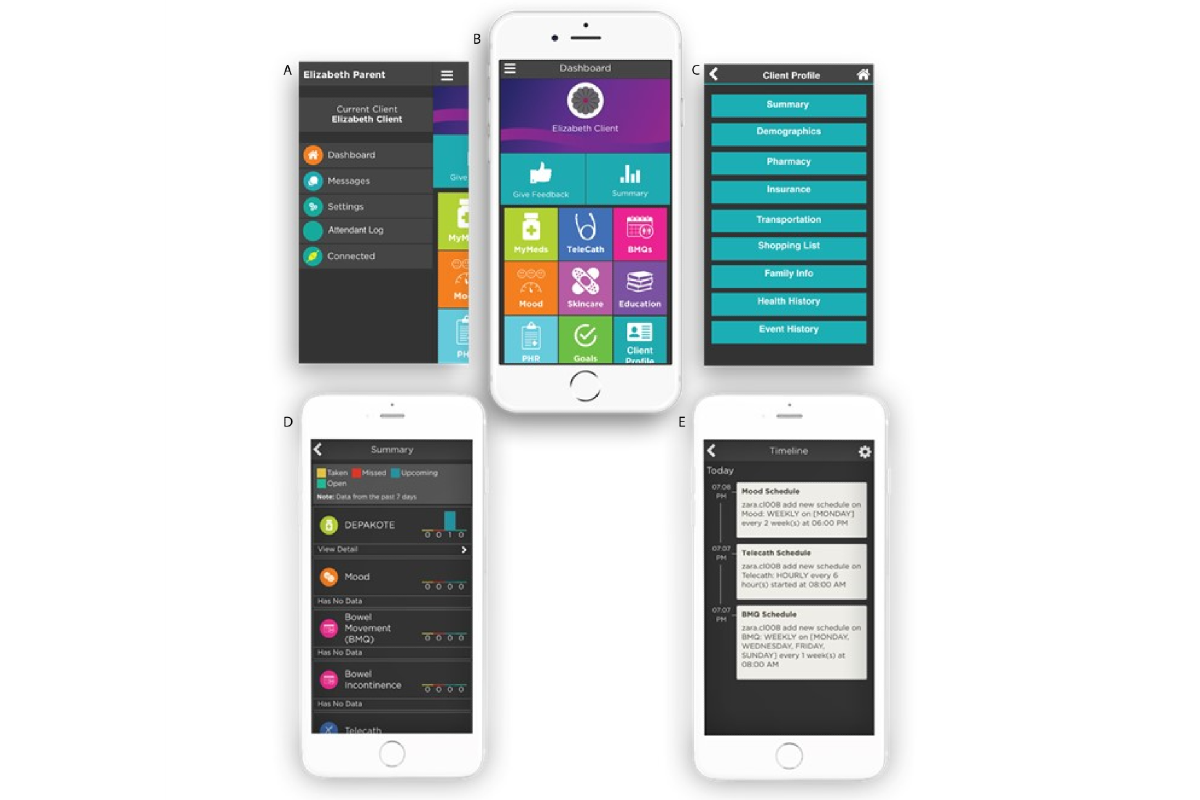
The caregiver app that allows attendants and other caregivers to monitor and provide feedback to their client/family member and access summary and timeline views.

The new caregiver app is designed to support a variety of common relationships between the caregiver and client (Figure 2). In previous iterations of the app, the functionality targeted only the unpaid (family) caregiver model. As shown in Figure 2A, the updated design of this app is now customizable to allow for meaningful use by both unpaid caregivers and paid caregivers. The new caregiver app includes tools that simplify the unpaid caregiver’s monitoring activities while also allowing access to client reports through each module (Figure 2B). For the paid caregiver, or attendant, the app retains monitoring and feedback functionality, permitting attendants to virtually track their clients’ activity as needed (Figure 2C-E).

### Monitoring or Tracking

CLASS suggested several monitoring or tracking changes to the app, which resulted in the creation of the Attendant Log module. Through free-text spaces and progress note areas in the Attendant Log module, attendants and caregivers track the progress of care provided to the client. Within the Attendant Log module, CLASS can electronically verify visits and track attendant activities such as “clocking-in” and “clocking-out” of sessions and completion of daily tasks.

**Figure 3:**
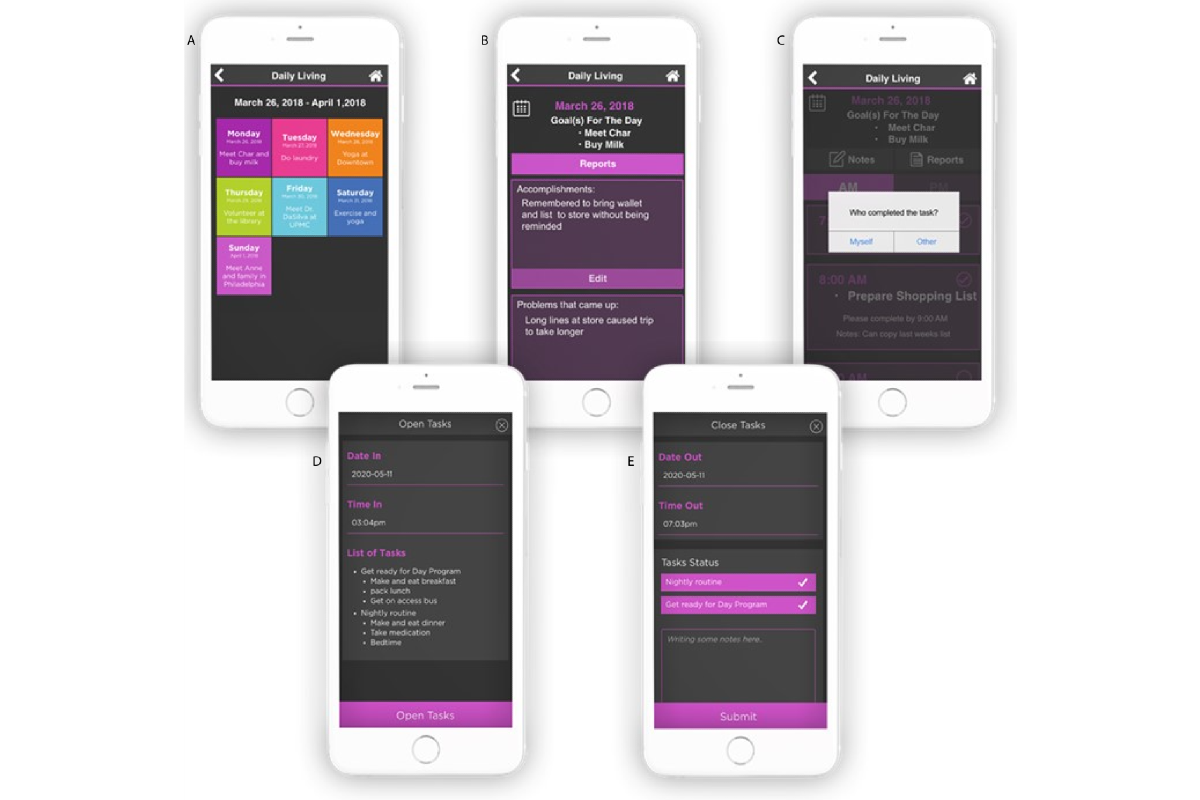
The Daily Living module that allows clients to see their upcoming events for the week. Daily goal reports display the client’s accomplishments and any encountered problems. Attendants can clock in and clock out, access task lists, and document completion of assigned tasks.

The Daily Living module, another addition created because of the focus group’s suggestions, provides both a weekly schedule and checklist for clients. Figure 3A shows the landing screen of the module, from which clients can view and edit their errands, training sessions, appointments, and other events for each day of the week. Clients can create goals for the day, enter notes, and view reports of their activity. As shown in Figure 3B, Goal reports list the client’s accomplishments and any encountered problems. Figure 3C shows that on completion of a task (eg, buying milk), the client is prompted to enter “who completed the task,” with options of selecting “myself” or “other.” “Other” typically refers to the client’s attendant because clients can specify difficult tasks for the caregiver to complete. These tasks appear on a checklist in the caregiver app. The attendant can record the day’s activities in the caregiver app, while the client can confirm and add notes. Caregivers can track tasks, and attendants can complete tasks within a module created in the caregiver app. Attendants can “clock in” and complete tasks as shown in Figure 3D-E. When attendants “clock out,” the client can confirm that tasks were indeed completed. This feature was developed because the focus group identified the need for improved attendant accountability and communication with clients. The goal of the client confirmation feature is to provide a second layer of authentication to information provided by the attendant about completion of tasks and time of arrival and departure.

**Figure 4:**
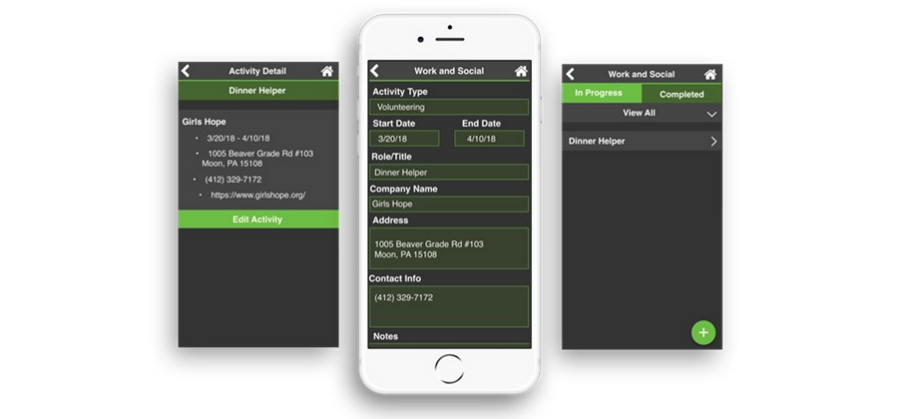
The Work and Social module that allows clients to enter details about work, social, and volunteer activities.

Within the newly created Work and Social module, clients can enter volunteer, work, and social activities along with start and end dates, roles or titles, company names, addresses, contact information, and notes. Clients mark their activities as “in progress” or “completed” (Figure 4).

**Figure 5:**
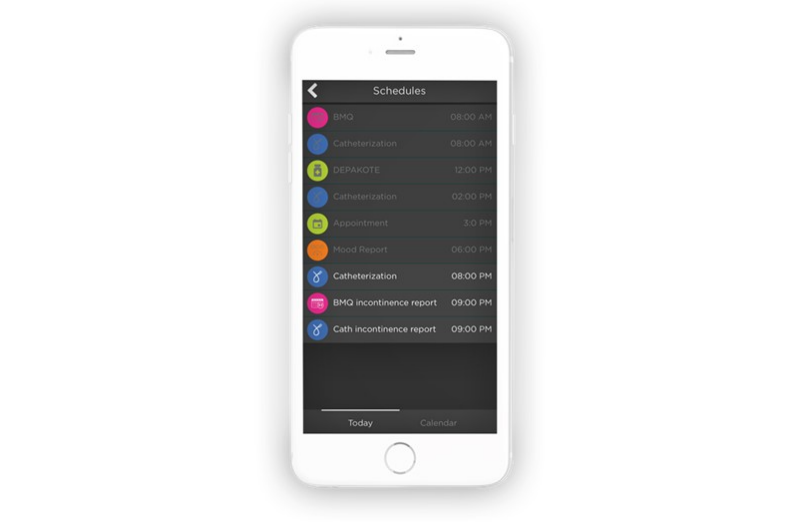
The calendar and schedule features that allow clients to track activities, appointments, and events.

CLASS recommended several monitoring and tracking changes to pre-existing modules. A medication adherence tracker was added to the My Meds module. The tracker allows caregivers and attendants to track client medication adherence. Now, through a centralized calendar (Figure 5), clients have access to reminders and medical and nonmedical appointments. Under the pre-existing Exercise module, clients can now enter their workouts and track their exercise progress and activity.

Through a future Incident Tracking module, clients will be able to report falls and related injuries. A Pain Tracker will also be included so that clients can document pain characteristics. It will also be possible to track attendant training from the portal. An electronic record documenting that the caregiver reviewed training information and complied with funding sources will automatically be created and stored in both the clinician portal and a private cloud. The system will also track what modules the attendant completed. Finally, clients will be able to track expenses and learn how to budget in a future finance module.

### Education

**Figure 6:**
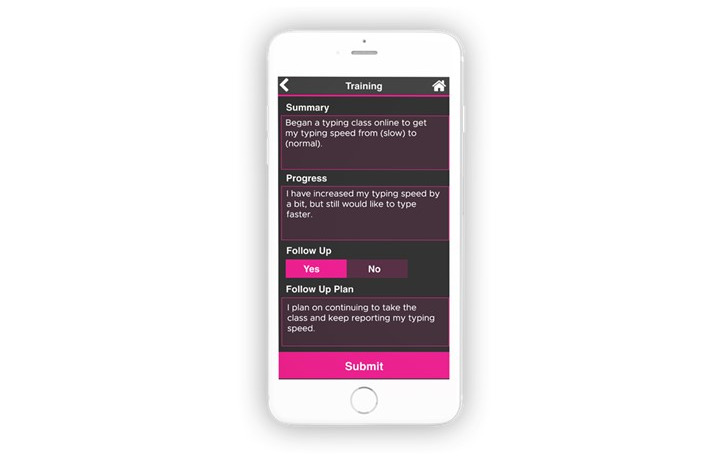
The Training module that allows clients to enter work-related and non–work-related training information.

CLASS requested the inclusion of a new Training module. The newly designed training module contains work-related and non–work-related training. Both active and past training are recorded. Within the module, clients can enter the job training location, addresses, and contact information (Figure 6). Clients can note their work progress and develop follow-up plans.

**Figure 7:**
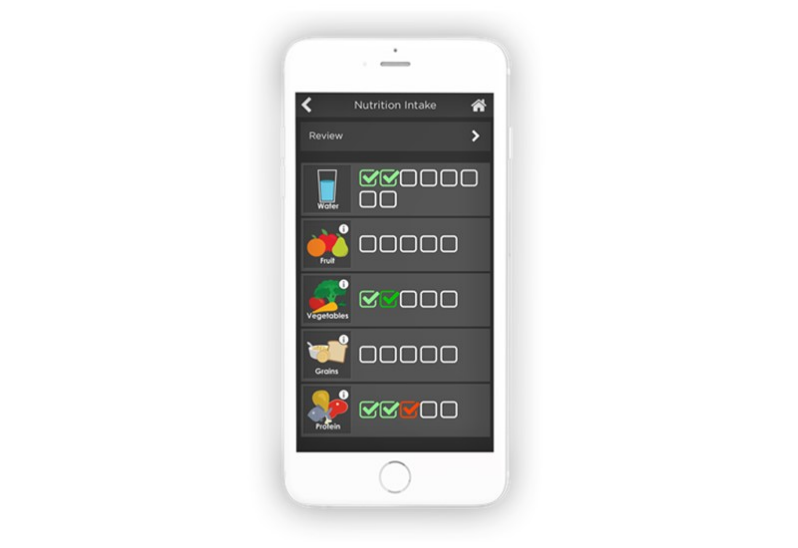
The Nutrition module provides nutritional advice and education.

CLASS also suggested updating the pre-existing Nutrition module (Figure 7) to provide more detailed diet information, including specific recommendations, such as recipes, to support healthy eating. In response to their comments, the future nutrition module will include recipe recommendations, a list of health foods, and information on various diets (eg, low-cholesterol diets, diets for patients with diabetes, gluten-free diets). Further, CLASS requested that certain education modules be available for caregivers and attendants. Through these modules, caregivers and attendants will be able to learn more about their clients’ conditions, train on the client’s care plan, and track client results of the quiz for content retention. These changes will be implemented in future versions.

The CLASS focus group also requested that a module be designed to provide wheelchair users with more information on wheelchair features, maintenance, and training. Within the wheelchair module, the guidebook was developed for clients to access educational information about power and manual wheelchairs. Under the manual wheelchair section, users can view a list of manual wheelchair components with corresponding images; for example, users can learn about frame types, cushion options, positioning accessories, and handrim designs. Users can also access information on how to ergonomically optimize their wheelchair through the set up and positioning of different components. The manual wheelchair skills section allows users to view videos of various wheelchair skills ranging from simple maneuvers to more advanced skills. Currently, the power wheelchair section allows users to view various component options for power wheelchairs through images and Graphics Interchange Format that illustrate the functionality of each component.

### Support

**Figure 8:**
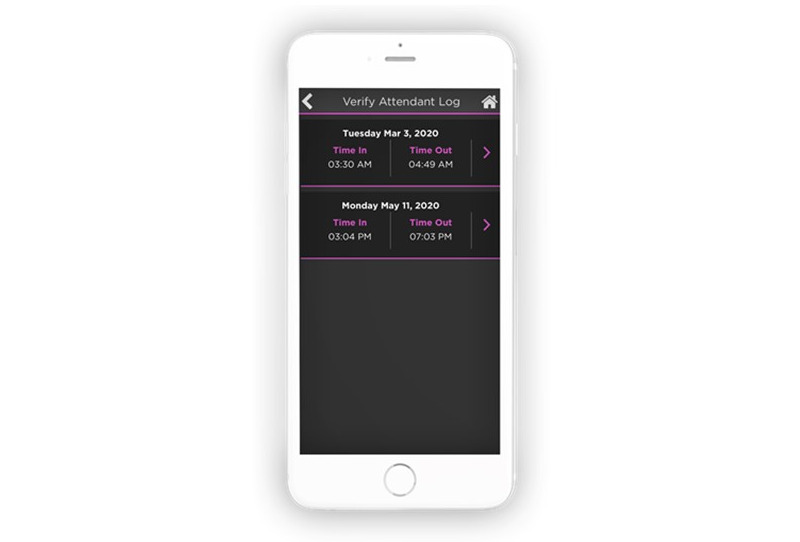
Clients can verify that an attendant provided care and completed tasks.

A module that allows clients to input feedback, such as the late arrival of an attendant, was also suggested. The Notes and Reports section within the new Daily Living module allows feedback input; after attendants complete their session, clients can accept or reject their report of completed tasks and note any issues (Figure 8).

CLASS requested changes to the pre-existing Goal Setting module for meaningful reporting of goal progress to funding organizations. The revised Goal Setting module provides users with a centralized area for goal creation. Here, clients are encouraged to include nutrition goals, such as healthy eating and physician-approved diets, as well as goals to increase their independence and community participation. Through the caregiver app, caregivers and service workers can ensure that clients are achieving their specified goals and view tracking data that quantifies goal progress. With respect to a future design criterion, clients will also be able to upload exercise photos to receive feedback and suggestions related to exercise.

### Privacy and Security

CLASS requested termination settings, such as the ability to remove the app from the client’s phone via the portal, which are now included under the Configuration module. Local information stored on the app can now be removed remotely via the portal. Additionally, caregivers can now access information about multiple clients through the Client Switcher module. This is useful for attendants who are usually assigned to multiple clients.

In the future, clients will be able to change their privacy settings to control what personal information is shared with the caregiver. Of note, the client app does not need to be paired with a caregiver app if the client does not have an assigned attendant. With respect to client app parameters, certain features will be locked. For example, it is possible to lock content such that a client is unable to delete medication or exercise reminders if that feature is required for a particular program.

### Reminders

**Figure 9:**
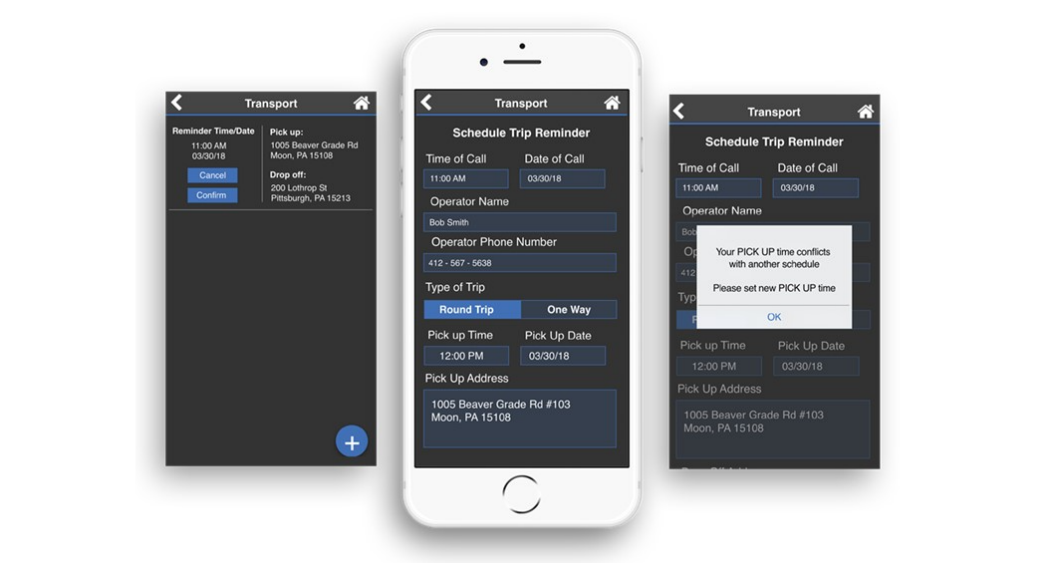
The Transportation module that allows clients to enter information pertaining to upcoming transportation.

Through the newly created Transportation module, clients now receive reminders to arrange for transportation after scheduling an appointment. Within the Transportation module, clients can enter a preferred time for a pick-up reminder call, date of the reminder call, operator name, operator phone number, type of trip (round trip or one way), pick-up time, pick-up date, pick-up address, and drop-off address. Clients receive a warning message if their specified pick-up time conflicts with another calendar activity (Figure 9). If transportation is added by a caregiver, clients must confirm the newly scheduled event. Clients can also cancel their transportation but must enter a reason for cancellation.

In the future, through a Global Reminder feature, clients will be periodically reminded of important agenda items, including ad-hoc prompts to report possible needs (eg, “Do you need to go to the grocery store this week?”). The Exercise module will push notifications that encourage exercise to the client. Finally, future design criteria will include “charge device” audio reminders as clients often forget to charge their devices.

CLASS noted that clients would benefit from a Supplies module that will allow users to track their medical supplies and set reminders for maintaining their stock. This module allows clients to enter information about their medical supplies such as type, purpose, vendor, quantity, and order date. In the Supplies module, clients can set reminders to order more supplies as needed. The app includes auto-fill supply options in addition to free-text functions. Future design criteria will allow for more automation, such as monthly reminders that alert users of the need to reorder a 30-day stock of supplies.

### Accessibility

CLASS requested text-to-speech and voice-command features. These requests were not implemented because Apple and Android devices contain built-in accessibility features. These features include “spoken speech/content” that allows users with limited vision to hear text, “voice commands” that allows users with impaired mobility to control their devices with verbal commands, and “switch access” that allows individuals with impaired mobility to control their device using a keyboard or a mouse. Further, iMHere 2.0 already contains accessibility features that allow clients to adjust text size, line height, button size, font style, button spacing, and hand preference. Mood scales consisting of emoticons instead of numbers will be implemented in a future build.

### Notes

**Figure 10:**
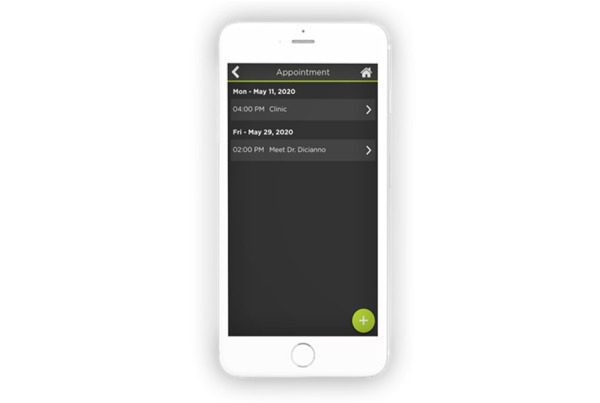
The calendar displays upcoming medical and nonmedical appointments.

The Appointment module is a newly created submodule within the PHR module. The Appointment module includes a calendar that displays upcoming medical and nonmedical appointments (Figure 10). By selecting the appointment, the client can view details such as date, time, and provider information (name, address, and phone). Additional fields for a summary of the appointment and notes are also included. The client can enter the appointment details by selecting “New Appointment.” Before submitting the form, the client must indicate whether they need to schedule transportation. If clients do not have enough information to complete the form in its entirety, they will receive reminders until the form is completed and submitted. Within the notes section, clients can create a checklist of medical condition–related questions to ask the physician during doctor appointments.

**Figure 11:**
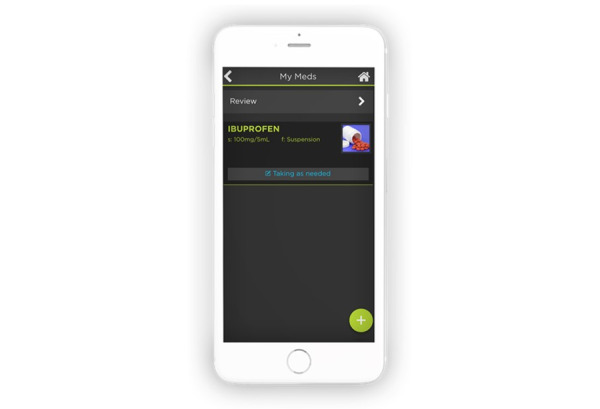
Pictures of pills and medication containers that allow clients to identify medications.

CLASS also recommended changes to the pre-existing medication module. To ensure that clients are taking the correct medications, CLASS asked that clients be able to upload pictures of pills and medication containers to the medication module (Figure 11), which was a feature that was added. Both the client and the caregiver also have access to a Medication Tracker that records client medication adherence.

With respect to future direction, clients will ultimately have access to a medical log feature within the pre-existing PHR module. The medical log will include vital signs (blood pressure, heart rate, body temperature, or respiratory rate), blood sugar readings, and height, weight, and body mass index measurements. To enter data, clients, caregivers, or clinicians will be able to add the date of the reading, the time, and the measurements (eg, systolic and diastolic pressures). Clients will eventually be able to view a graphical representation of their readings for a single day, week, month, or entire year.

### Safety

An emergency contact can be added from the phone’s lock screen. In Case of Emergency (ICE) contacts are accessible in the client profile. By selecting ICE, clients can retrieve contact information for their provider, support staff, and insurance company. The ICE module also lists restrictions such as medications or procedures that should be avoided during a medical emergency. Under “My Profile,” clients can also find advance directives, additional emergency contacts, and a list of providers and medications. QR code availability in future will allow users to scan their medications to confirm that the correct medication is administered.

### Profile

**Figure 12:**
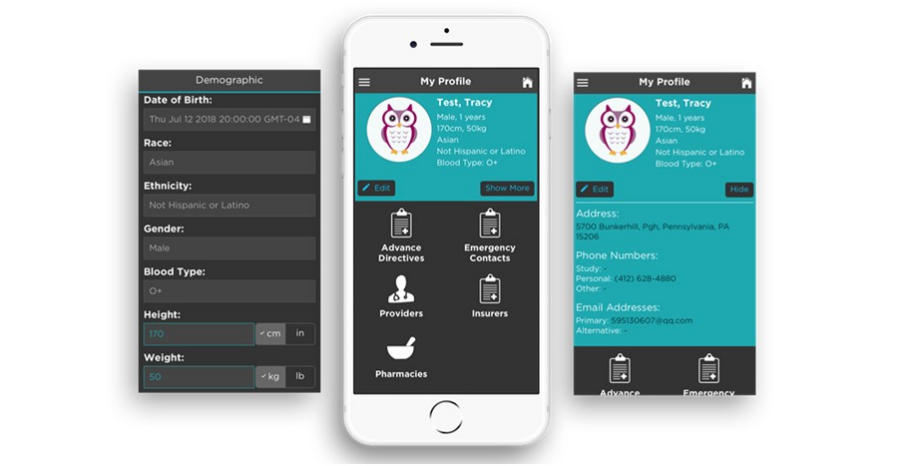
Clients can enter their contact information, demographics, emergency contacts, advanced directives, insurers, providers, and pharmacies.

CLASS suggested changes within the PHR module. The focus group proposed that staff could better serve clients if identifying client information was added to the PHR module. Within the revised module, clients can create and view a customized user profile. Clients can enter their gender, age, height, weight, race, ethnicity, and blood type (Figure 12). Clients can also update their profile to include information about likes, dislikes, and other introductory information such as pets at home, smoking status, and medical conditions, which can be then pushed to the attendant. If a client smokes or has a pet at home, the attendant will receive a digital alert.

### Overall Development Themes

Stakeholders emphasized that the revised iMHere 2.0 should remain client-driven to encourage self-sufficiency. They also stated that the system should be useable by the client alone or with a variety of different support roles from caregivers, attendants, and care managers. The original iMHere system 2.0 was designed as an adaptive system that allows clients to receive modules and content only as needed. The stakeholders felt it should continue to provide personalization of modules such that different clients could have different profiles and app configurations depending on their unique needs.

## Discussion

### Principal Findings

This study demonstrates how stakeholders within a community-based organization that provides services to individuals with disabilities can influence and shape the design of mHealth technology and how these designs can be improved to better serve individuals with disabilities.

The literature on chronic care management shows that promoting partnerships between recipients of services and the providers of the services has positive outcomes, especially when such partnerships facilitate self-management [20,21]. Important self-management skills include problem solving, decision-making, resource utilization, and goal-setting [21]. Thus, innovative systems to support individuals with chronic conditions and disabilities should not only incorporate features that empower users with tools for self-management but also facilitate sustainable partnerships.

mHealth systems are a potential way to deliver such support and empowerment. Some community-based organizations have expressed the need for dashboards that show live data to facilitate care decisions in real time [22] and allow users to record data “on the go” as a way to improve quality metric reporting [23]. An ideal software system could show the interrelationships among an individual’s medical problems, daily schedule, medical and social care plans, and goals [22]. Solutions are also needed to allow care providers to recognize red flag symptoms and intervene quickly, manage medications, and coordinate care while integrating the support of family and other caregivers of individuals with chronic conditions and disabilities [24].

To our knowledge, iMHere 2.0 is the only mHealth system designed to meet these needs and designed iteratively in partnership with the organization it serves. By gathering qualitative data from a focus group, this study was able to identify ways to re-design mHealth technology to address the needs of an organization supporting individuals with disabilities living in the community. This partnership resulted in a highly fruitful development process that led to major advances in the mHealth system. Additionally, our team generated design criteria to be utilized in future iterations of the system.

Some limitations of our development process, however, deserve discussion. First, iMHere 2.0 was not designed to meet the needs of all community-based organizations or users. Concurrent work is being carried out to further refine iMHere 2.0 to support other organizations with different workflows, users, and organizational structure. Second, although we attempted to incorporate all the necessary changes suggested by CLASS, some suggestions could be met with native features of phones or tablets. For instance, low–battery-level reminders, text-to-speech or read-out-loud features, and voice-command features exist. Because these may not meet the needs of all users, future accessibility advancements are needed for users with specific accessibility needs.

### Future Work

Ongoing work is being conducted to assess the usability and feasibility of iMHere 2.0 and to study the impact of augmenting programs offered at CLASS with iMHere 2.0 to measure the impact on client outcomes such as community integration. We are also conducting implementation research with other community-based organizations, including those that provide attendant care services in the home, to identify facilitators and barriers to wide-scale implementation of the technology. In parallel, we will continue development that is inspired by design criteria generated from each of our studies. We also aim to integrate some features of iMHere into the electronic health record system to reduce data entry requirements and facilitate data sharing.

### Conclusions

Individuals with disabilities can thrive in community settings when they are given access to high-quality health care, supportive caregivers, and community support. Using an iterative design process in partnership with a community-based organization, we built an mHealth system with new features to support community integration of individuals with disabilities.

## Appendix

Multimedia Appendix 1 The requested new features for iMHere 2.0.

## Abbreviations

CLASS: Community Living and Support Services
CPP: Community Partners Program
ICE: In Case of Emergency
mHealth: mobile health
PHR: Personal Health Record
