# Supplementary material for: Mobile Health to Support Community-Integration of Individuals With Disabilities Using iMHere 2.0: Focus Group Study
Source: JMIR Hum Factors. 2022 Mar 4;9(1):e31376. doi: 10.2196/31376 (PMC8933796; doi:10.2196/31376)
Supplement: Multimedia Appendix 1 [file humanfactors_v9i1e31376_app1.docx]

| Theme | Requested features | Reasoning | Priority | Implementation |
| --- | --- | --- | --- | --- |
| **Monitoring** | | | | |
|  | Free-text areas and space for progress notes for the attendant or caregiver | Progress notes allow the attendant or caregiver to track the progress of care provided to the client. Further, the client can review and sign progress notes, verifying that care was provided. Free text areas allow for additional notes about clients and visits, with the aim of improving future care. | Necessary | New feature (Attendant Log module) |
|  | Electronic visit verification | CLASS needs to verify that an attendant visit occurred. | Necessary | New feature (Attendant Log module and Portal) |
|  | Reminders to “check in” | On occasion, attendants forget to “clock in.” | Necessary | New feature (Attendant Log module and Portal) |
|  | Care manager access to data | Care managers must be able to access data from clients and caregivers. | Necessary | Existing feature (Portal) |
|  | Tracking falls | Falls are notoriously challenging to report. | Desired | Future design criterion |
|  | Pain tracker | Clients experience both acute and chronic pain. | Desired | Future design criterion |
|  | Expense log | By inputting spending information, clients can learn how to maintain a budget. | Desired | Future design criterion |
|  | Client and caregiver generated checklist for daily tasks | Clients occasionally need to assign tasks to a caregiver or attendant while learning to develop skills in directing their own care. | Necessary | New feature (Daily Living module) |
|  | Tracking of Daily Living Checklist by the attendant or caregiver | Attendants and caregivers need a way to track the tasks that the client wishes them to perform. | Necessary | New feature (Daily Living and Attendant Log modules) |
|  | Medication adherence tracker | Caregivers or attendants need to track if clients are adhering to their medication regimen. | Necessary | Existing feature  (MyMeds module) |
|  | Ability to find, schedule, and make notes on volunteer, work, and social plans | Clients need support to improve participation in social and volunteer opportunities. | Necessary | New feature (Work and Social module) |
|  | Tracking of the training activity of the caregiver or attendant | A checklist for caregiver or attendants’ completed training activities. Reports can be generated for certification purposes. | Desired | Future design criterion |
|  | Calendar | Clients need a calendar to manage personal and medical activities, tasks, and events. | Necessary | Existing feature (Dashboard, Schedule) |
| **Education** | | | | |
|  | Specific education module for caregiver or attendant use | A library should be built with training or education modules for caregivers to learn more about their clients’ conditions. | Desired | Future design criterion |
|  | Track clients’ quiz performance | Caregivers or attendants should be able to track clients’ quiz results to see if they learned information provided to them. | Desired | Future design criterion |
|  | Client interaction education | An education module on how to interact with clients should be developed. | Desired | Future design criterion |
|  | Care plan training | Caregivers must be trained on the client’s care plan. | Desired | Future design criterion |
|  | Nutrition for specific diets | Clients need information about specific diets (eg, diets for patients with diabetes, low cholesterol diets). | Desired | Future design criterion |
|  | Recipes | Clients need recipe recommendations for specific diets. | Desired | Future design criterion |
| **Support** | | | | |
|  | Nutrition goals | Clients need motivation to eat well. | Necessary | Existing feature (Goal Setting) |
|  | Goal assistance | Caregivers assist clients with achieving goals such as exercising. | Necessary | Existing feature (Goal Setting) |
|  | Recommended foods | Clients need recommendation on foods to eat to maintain health. | Desired | Future design criterion |
|  | A place to document complaints | This feature would allow clients to report issues such as when a transportation service arrives late and, as a result, the client misses an appointment. | Necessary | New feature (Notes and Reports sections within the Daily Living module) |
|  | Option to upload exercise photos and tracking function | Clients could benefit from receiving feedback and suggestions related to exercise (eg, feedback on form during exercise). | Desired | Future design criterion |
| **Privacy and security** | | | | |
|  | Client privacy settings | Clients should be able to control what personal information is shared with caregivers. Share settings should include “view,” “view and edit,” or “no access.” | Desired | Future design criterion |
|  | Role-based access for caregivers | Caregivers need to access information about multiple clients. | Necessary | Existing feature (Client Switcher) |
|  | Termination settings | The username and password should be able to be turned off or detached from the client account on termination. It would be important to remotely deactivate the app from the client’s phone if the device is stolen or misplaced. | Necessary | Existing feature (Configuration) |
|  | Remove “delete” ability | Consumers should be able to revert parameters that are inadvertently altered to the original settings. Clients should not be able to remove important reminders such as exercise and medication notifications. | Desired | Future design criterion |
| **Reminders** | | | | |
|  | Free text reminders | Clients often forget to complete tasks associated with mail or phone calls, such as paying bills, registering for events, and completing paperwork. | Desired | Future design criterion |
|  | Ad-hoc prompts for clients to report possible wants or needs | It should be possible to add extra reminders for clients, eg, “Did you want to buy anything this week?” | Desired | Future design criterion |
|  | Activity tracker or reminder | Some clients struggle to stay physically active or find motivation to exercise. | Desired | Future design Criterion |
|  | A way to record and remind clients about transport for medical and nonmedical appointments | Clients need to record the transportation needed for appointments or other activities. Medical transportation typically must be scheduled 1 day in advance, while nonmedical transportation is often scheduled 2 days to 2 weeks in advance. A push notification should remind clients about upcoming transportation scheduling deadlines. | Necessary | New feature (Transportation module) |
|  | Charge device audio reminder | Clients often forget to charge their devices. | Desired | Future design criterion |
| **Accessibility** | | | | |
|  | Accessibility for limited manual dexterity | A voice-command feature is needed for clients with limited manual dexterity. | Desired | Future design criterion |
|  | Allow users to send images of skin with minimal prompting | Those with cognitive impairment may find it difficult to answer questions. | Necessary | Existing feature (Skincare module) |
|  | Mood scales using emoticons as opposed to numbers | Images are often easier to understand for clients. | Desired | Future design criterion |
|  | Text-to-speech or read-out-loud feature | A text-to-speech feature would provide clients an alternative to reading written text. | Desired | Future design criterion |
| **Notes** | | | | |
|  | Description of medical appointment | Often, clients forget why a medical appointment was scheduled and the type of specialist they are scheduled to meet. | Necessary | Existing feature (PHR^a^ and notes section of Appointment module) |
|  | Checklist of questions to ask the doctor | Clients often forget to ask important questions. | Necessary | Existing feature (PHR and notes section of Appointment module) |
|  | Summary of appointment | Clients can reference the content discussed at their medical appointments. | Necessary | Existing feature (PHR and notes section of Appointment module |
|  | Note taker | Clients need a feature dedicated to note taking. | Necessary | Existing feature (PHR and notes section of Appointment module |
|  | A photo of a pill or pill container to be included with the medication reminder | This feature may help to ensure that a client is taking the correct medication. | Necessary | Existing feature (MyMeds module) |
| **Safety** | | | | |
|  | QR code capability for prescriptions | This may help to ensure that a client is taking the correct medication. | Desired | Future design criterion |
|  | Emergency contact | Users need to identify an emergency contact that should be accessible from the phone’s lock screen. | Necessary | Existing feature (My Profile) |
| **Profile** | | | | |
|  | Information about clients available to attendants that includes information about likes, dislikes, and other introductory information | Caregivers will be able to build a more genuine relationship with clients if they have information about clients’ interests. Further, it is important for the caregiver to know for allergy reasons if the client has pets or smokes. The caregiver can also obtain more information about the client’s health condition and nutritional needs. | Necessary | New feature (Client profile summary) |
|  | Insurance information | Clients can specify their insurance provider for self-review and for review by others. | Necessary | Within existing module  Existing feature (Client Profile) |

^a^PHR: Personal Health Record
